# Deep-sea bacteria trigger settlement and metamorphosis of the mussel *Mytilus coruscus* larvae

**DOI:** 10.1038/s41598-020-79832-8

**Published:** 2021-01-13

**Authors:** Rui-Heng Chang, Li-Ting Yang, Ming Luo, Yihan Fang, Li-Hua Peng, Yuli Wei, Jiasong Fang, Jin-Long Yang, Xiao Liang

**Affiliations:** 1 International Research Center for Marine Biosciences, Ministry of Science and Technology, Shanghai Ocean University, Shanghai, China; 2 Southern Marine Science and Engineering Guangdong Laboratory, Guangzhou, China; 3 Hainan Academy of Ocean and Fisheries Sciences, Haikou, China; 4 Hadal Science and Technology Research Center, Shanghai Ocean University, Shanghai, 201306 China

**Keywords:** Ecology, Ecology

## Abstract

Bacteria from coast seawaters are widely known to induce larval recruitment of many invertebrates. However, whether and how deep-sea bacteria, that play crucial roles in the ecological and biogeochemical cycles, promote larval recruitment remains little known. Here, the interaction between deep-sea bacterial biofilms (BFs) and *Mytilus coruscus* larvae was tested. All these nine deep-sea bacterial isolates triggered planktonic-sessile transition, and the highest percentage of post-larvae was observed in *Virgibacillus* sp. 1 BF. Except for *Pseudomonas* sp. 3, *Pseudoalteromonas* sp. 32 and *Bacillus* sp. 13, other BF cell  densities were significantly related to their corresponding inductive efficiency. The deep-sea *Virgibacillus* sp. 1 BFʼs cue that triggers planktonic-sessile transition was uncovered. Treating *Virgibacillus* sp. 1 BFs through physic-chemical approaches reduced inducing impact and cell survival. The conditioned water collaborated with formalin-fixed *Virgibacillus* sp. 1 BF hoisted planktonic-sessile transition efficiency in comparison to each one alone. Thus, two signals derived from deep-sea bacteria trigger planktonic-sessile transition in *M. coruscus*. This finding firstly demonstrates that deep-sea bacteria has good potential for application in the mussel seed production and provides novel insight to clarify the bacteria-mussel interaction.

## Introduction

*Mytilus coruscus* is an economically important species of mussel in China^1^, which is widely distributed in coastal areas. Similar to most marine invertebrates, larvae of *M. coruscus* undergo a stage of planktonic and benthic during growth and development. During the planktonic stage, larvae will find suitable settlement bases and complete the process of settlement and metamorphosis before they grow into adults^2^. However, because of the decrease of natural resources of *M. coruscus* and the shortage of artificial cultivation technology, the development of *M. coruscus* breeding industry has been restricted^3^. Thus, the development of seeding production technology of *M. coruscus* has become an urgent matter.

During the hatchery of *M. coruscus*, settlement and metamorphosis are essential for larval survival and development, as well as key for successful breeding. Biofilms (BFs) played a vital role in settlement process of benthic animals, such as mussels, oysters, and sea urchins^4–9^. In *M. coruscus*, natural BFs^10^ and offshore bacteria^11–13^ could promote the settlement and metamorphosis process in this species. Therefore, screening bacteria with inducing activity from novel resources becomes a meaningful work in mussel aquaculture.

The deep sea receives global attention as one of the most important microbial-driven ecosystems, and microbes in deep sea play a vital role in ecological and biogeochemical cycles^14,15^. The number of species and the amount of microorganisms in the deep sea are much greater than previously thought^16^. However, whether and how the deep-sea bacteria trigger larval development of commercially marine molluscs remains little known. Here, the authors clarified (1) whether deep-sea bacteria trigger planktonic-sessile transition in *M. coruscus*, and (2) which characteristics of inductive cues of the deep-sea bacteria act on planktonic-sessile transition. The purpose was to explore the possibility to the application of deep-sea bacteria on the aquaculture of *M. coruscus* and clarify the mechanism of planktonic-sessile transition in marine bivalves.

## Results

### Induction of deep-sea bacterial BFs

**Figure 1:**
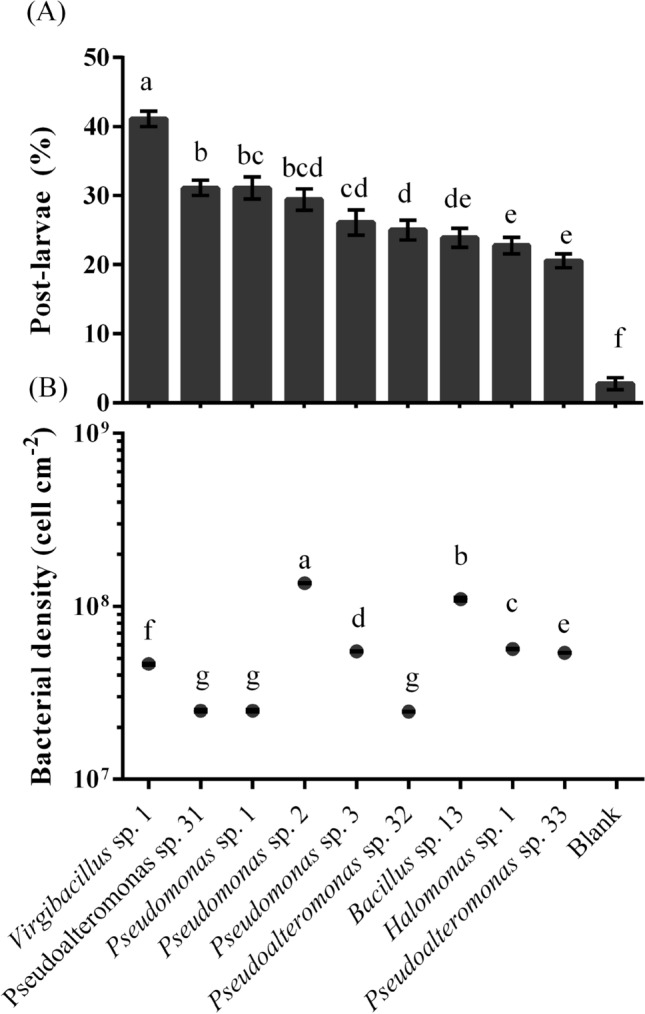
*M. coruscus* post-larval rates (**A**) in deep-sea bacterial BFs in relation to BFʼs cell density (**B**). Small letter suggests significant variance (*p* < 0.05). Means ± SE (n = 9).

The information of deep-sea bacteria used in the experiment is shown in Table 1. Compared with the blank group, all BFs of tested strains showed an inductive effect in the settlement and metamorphosis (*p* < 0.05, Fig. 1A) and no mortality was observed. Genera *Virgibacillus*, *Pseudoalteromonas* and *Bacillus* isolated from deep-sea sediments showed strong or moderate inducing activity (Fig. 1A). Genera *Pseudomonas*, *Pseudoalteromonas* and *Halomonas* isolated from the deep-sea water showed moderate inducing activities (Fig. 1A). Among all tested deep-sea bacteria, *Virgibacillus* sp. 1 isolated from Shimokita Peninsula had the highest inducing activity. The results showed that the level of inducing activity was related to the strain itself.

**Table 1:** 16S rDNA gene sequence analysis and basic information of the deep-sea bacterial strains.

Isolate	Accession no	BLAST closest match	Accession no of closest match	Similarity %	Location	Depth	Longitude	Latitude	Isolated source	Isolation time
6R	MK389412	*Virgibacillus* sp. 1	CP017762	99	Shimokita Peninsula	1180 m	142.20°E	41.18°N	Sediment	2013.03
J-12	MK368449	*Pseudoalteromonas* sp. 31	CP011041	99	New Britain Trench	5000 m	149°46.613′ E	06° 59.548′ S	Sea water	2016.09
ZNW	MK368450	*Pseudomonas* sp. 1	HM224410	99	New Britain Trench	8900 m	153.83°E	6.36°S	Sea water	2017.01
NBT0603	MK368451	*Pseudomonas* sp. 2	EU603457	99	New Britain Trench	8900 m	153.83°E	6.36°S	Sea water	2017.01
DT5000-1	MK368453	*Pseudomonas* sp. 3	MH169261	99	Mariana Trench	5000 m	142.36°E	11.43°N	Sea water	2017.01
M7NS11	MK368455	*Pseudoalteromonas* sp. 32	NR116629	99	New Britain Trench	4524 m	149°45.534′ E	06° 40.842′ S	Sediment	2016.09
M7WS21	MK368456	*Bacillus* sp. 13	KC764988	99	New Britain Trench	3908 m	149°45.462′ E	06° 36.894′ S	Sediment	2016.09
M4	MK368457	*Halomonas* sp. 1	EU660349	99	New Britain Trench	6700 m	149°46.613′ E	06° 59.548′ S	Sea water	2016.09
M7E11	MK368458	*Pseudoalteromonas* sp. 33	KT036405	99	New Britain Trench	1000 m	151°58.5042′E	06°02.1243′S	Sea water	2016.09

The bacterial density of nine deep-sea bacterial BFs (initial bacterial density was 5.0 × 10^8^ cells ml^−1^) is as shown in Fig. 1B. At the same initial bacterial density, there were significant differences in the density of BFs formed by bacteria from different sources (*p* < 0.05) (Fig. 1B). *Virgibacillus* sp. 1 BFs exhibited the highest post-larvae rates (Fig. 1A) when bacterial density was 4.6 ± 0.04 × 10^7^ cells cm^−2^. *Pseudoalteromonas* sp. 31, *Pseudomonas* sp. 1 and *Pseudoalteromonas* sp. 32 were lower than the bacterial density of *Pseudomonas* sp. 2, whereas four deep-sea bacteria showed similar inducing activity (Fig. 1). *Virgibacillus* sp. 1 had a higher inducing activity than *Pseudomonas* sp. 2, while its BF cell density was lower than *Pseudomonas* sp. 2 (Fig. 1).

**Figure 2:**
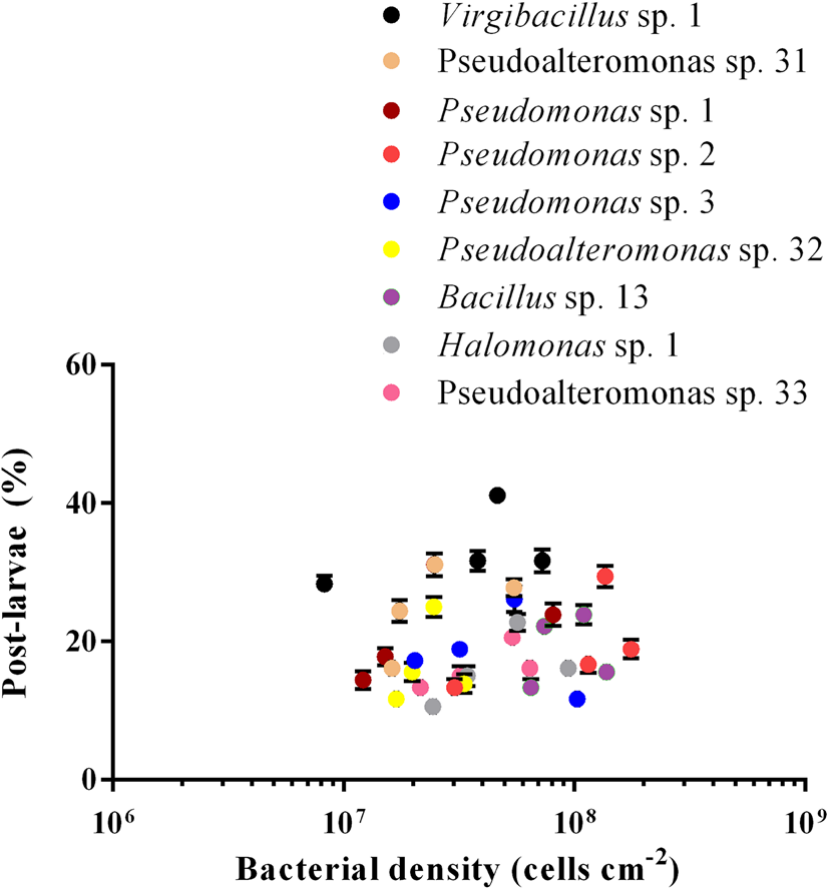
*M. coruscus* post-larval rates on deep-sea bacterial BFs under dynamic changing densities. Post-larvae rate: Means ± SE (n = 6); bacterial densities: Means ± SE (n = 10).

**Figure 3:**
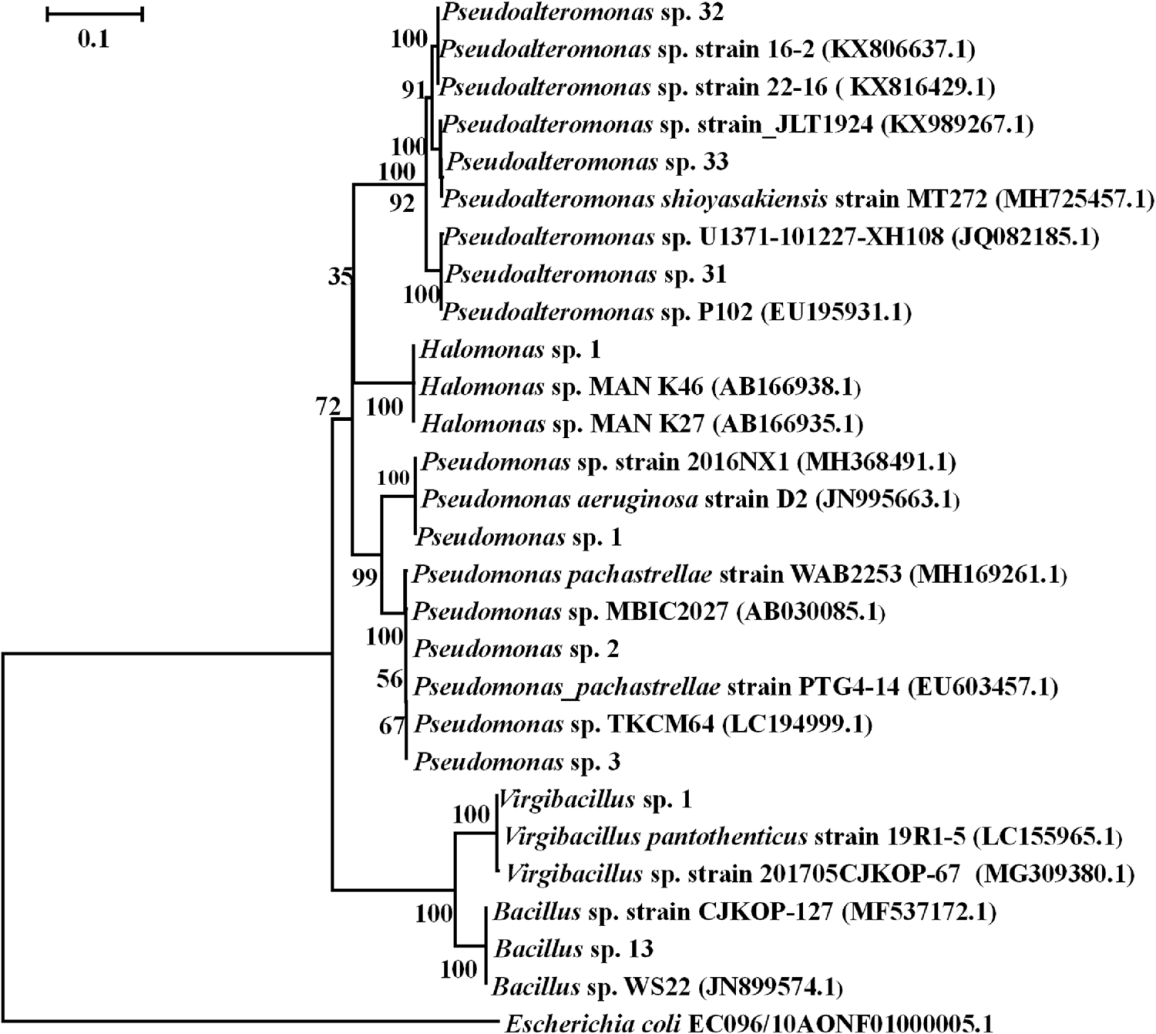
Phylogenetic tree of 16S rDNA gene sequences from tested deep-sea bacteria through Neighbor-Joining method and Jukes–Cantor model. Scale bar = 0.1 substitutions per nucleotide position.

The percentage of post-larvae in *Virgibacillus* sp. 1 increased first and then decreased with the increase in bacterial density (Fig. 2). When the bacterial density was 4.6 ± 0.04 × 10^7^ cells cm^−2^, the percentage of post-larvae in *Virgibacillus* sp. 1 was the highest. Bacterial density had a positive correlation with the inductive effect (*p* < 0.05), except for *Pseudomonas* sp. 3, *Pseudoalteromonas* sp. 32 and *Bacillus* sp. 13 (Table 2). The correlation was moderate (0.8 > r > 0.6) for *Pseudoalteromonas* sp. 31 and *Pseudomonas* sp. 1, and weak (r < 0.6) for *Virgibacillus* sp. 1, *Halomonas* sp. 1, *Pseudomonas* sp. 2, and *Pseudoalteromonas* sp. 33. Different methods and models were used for phylogenetic analysis and similar results were obtained (Fig. 3). *Halomonas* sp. 1 and *Pseudoalteromonas* sp. 33 belonged to different genera and exhibited the similar inductive effect (Fig. 1A). On the contrary, *Pseudoalteromonas* sp. 31 and *Pseudoalteromonas* sp. 33 belonged to the same genus exhibited different inductive effect (Fig. 1A). This indicates that species of bacteria may have no direct relationship with inducing ability.

**Table 2:** Correlation analysis between BF cell density of deep-sea bacteria and inductive efficiency. r = Spearman’s rank order correlation analysis; *p*: *p*-value; *: significant at *p* < 0.05.

Tested bacteria	Bacterial density	
	r	*p*
*Virgibacillus* sp. 1	0.3624	0.0298*
*Pseudoalteromonas* sp. 31	0.6822	< 0.0001*
*Pseudomonas* sp. 1	0.6599	< 0.0001*
*Pseudomonas* sp. 2	0.5027	0.0018*
*Pseudomonas* sp. 3	− 0.2543	0.1345
*Pseudoalteromonas* sp. 32	0.2951	0.0806
*Bacillus* sp. 13	0.1474	0.3910
*Halomonas* sp. 1	0.5389	0.0007*
*Pseudoalteromonas* sp. 33	0.3842	0.0207*

### Effects of deep-sea *Virgibacillus* sp. 1 BFs treated

**Figure 4:**
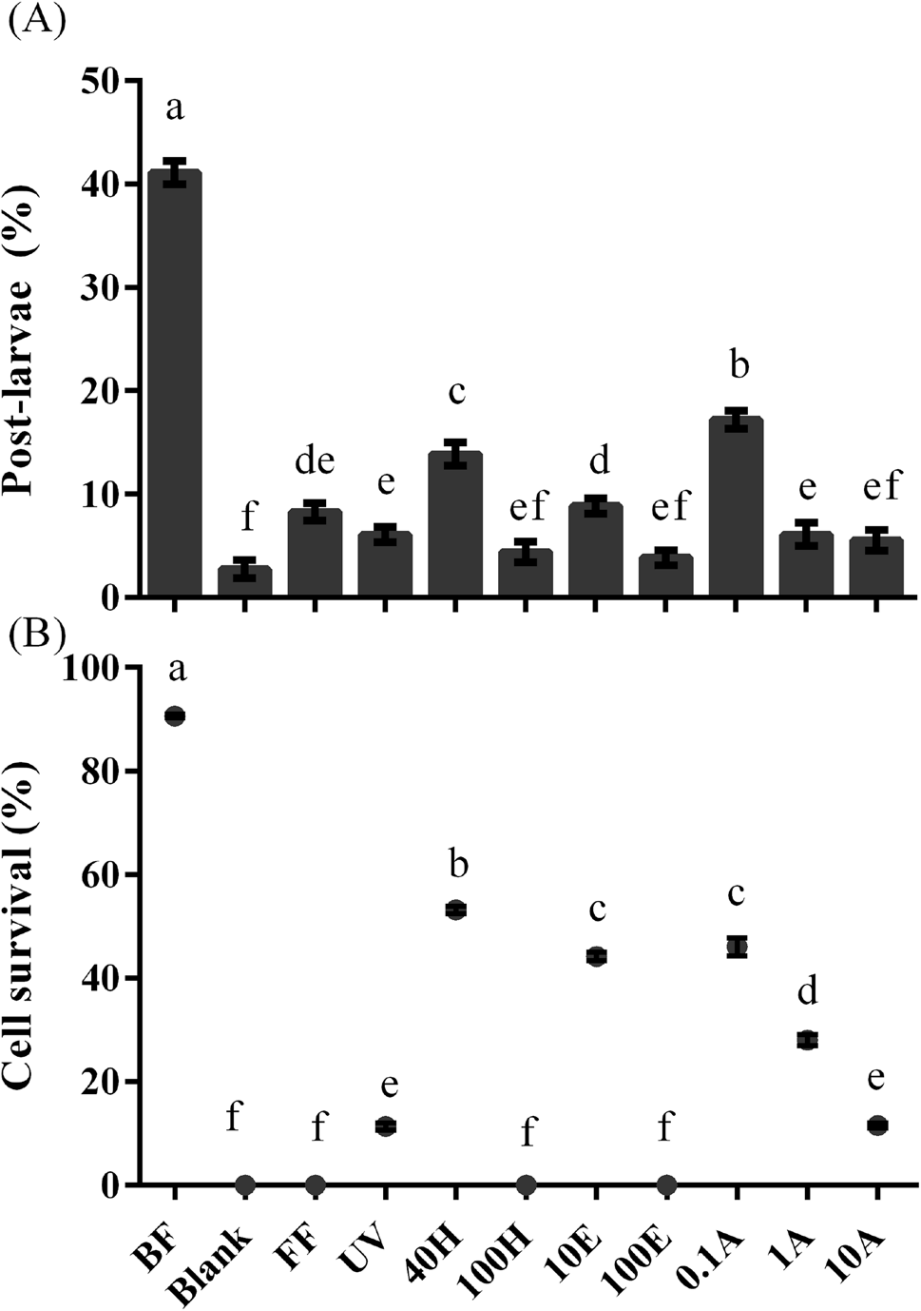
Post-larval rate (**A**) and cell survival (**B**) of deep-sea *Virgibacillus* sp. 1 BFs treated. Small letter suggests significant variance (*p* < 0.05). Post-larval rate: Means ± SE (n = 9); cell survival: Means ± SE (n = 10).

The *Virgibacillus* sp. 1 BF with the highest inducing activity (41 ± 1%) was treated, and the percentage of post-larvae (Fig. 4A) and bacterial survival (Fig. 4B) decreased significantly on treated BFs (*p* < 0.05). The percentage of post-larvae decreased significantly with the treatment of temperature and ethanol concentration (*p* < 0.05, Fig. 4A). The percentages of post-larvae were 10–20% on BFs treated by 40H and 10E (Fig. 4A). BF treated by 100H and 100E had no inducing activity (*p* > 0.05, Fig. 4A). With the increase of antibiotic concentration, the BF inducing activity decreased significantly (*p* < 0.05, Fig. 4A). The bacterial survival ranged from 40 to 60% after 10E and 0.1A treatment, while no bacteria survived after 100H, 100E, and FF treatment (Fig. 4B).

### The activity of conditioned water (CW) of the deep-sea *Virgibacillus* sp. 1 BF

**Figure 5:**
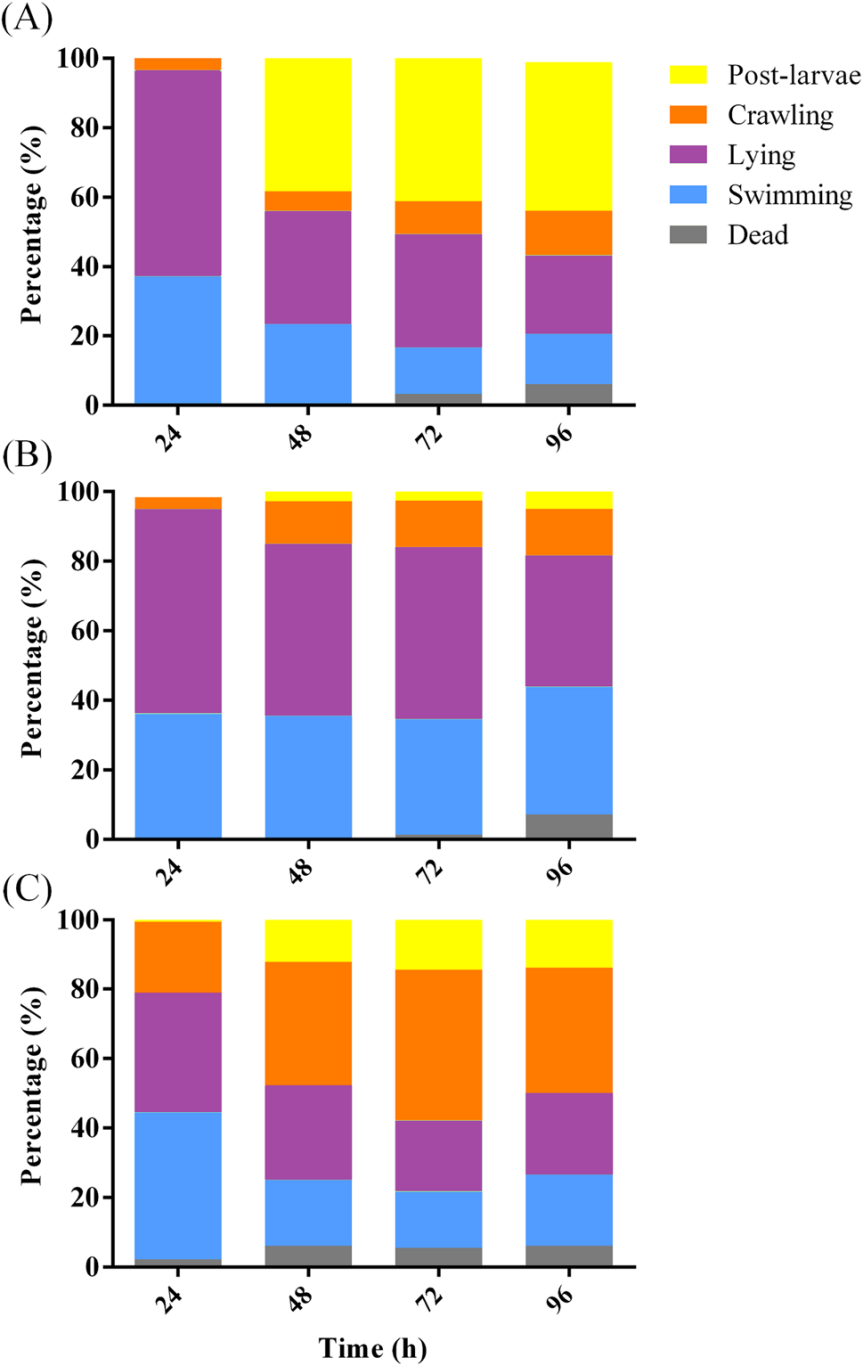
Behavior change of mussel larvae exposed to deep-sea *Virgibacillus* sp. 1 BF (**A**), AFSW (**B**), and CW of *Virgibacillus* sp. 1 BF. Means (n = 9).

In presence of *Virgibacillus* sp. 1 BFs, larvae of more than 80% were mainly swimming and lying at 24 h, and larval metamorphosis rate reached the highest at 48 h (Fig. 5A). In AFSW, more than 80% of the larvae were swimming and lying at 24 and 48 h (Fig. 5B). The percentages of crawling larvae in CW increased significantly comparing to that in AFSW and BF (*p* < 0.05, Fig. 5C). However, the post-larval rate in CW was significantly lower at 48 h than in the *Virgibacillus* sp. 1 BFs (*p* < 0.05). It indicated that CW had a significant effect on the larval crawling behavior but had little effect on metamorphosis.

### Synergistic effect of CW and FF of deep-sea *Virgibacillus* sp. 1 BF

**Figure 6:**
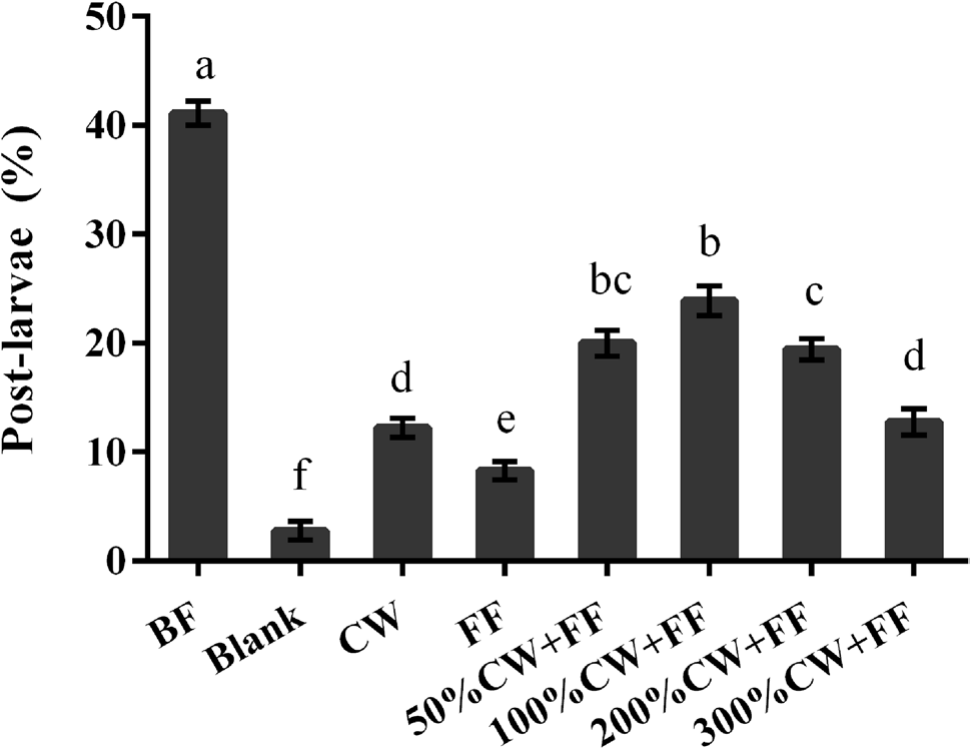
Post-larvae rate in CW and FF *Virgibacillus* sp. 1 BFs. Small letter suggests significant variance (*p* < 0.05). Means ± SE (n = 9).

CW (100%) as well as FF showed low inducing activity (Fig. 6). Except for 300%CW + FF, Higher post-larvae rate in CW + FF (50%CW + FF; 100%CW + FF; 200% CW + FF) was observed than that in FF or CW alone (*p* < 0.05, Fig. 6). With the increase of CW concentration, the inductive effect of CW + FF increased first and then decreased (Fig. 6); and the inductive effect was the highest at 100% CW + FF (Fig. 6). It indicated that there was a threshold for CW concentration.

### Confocal laser scanning microscopy (CLSM) images and biovolumes of extracellular polymeric substances (EPS) of deep-sea bacterial BFs

**Figure 7:**
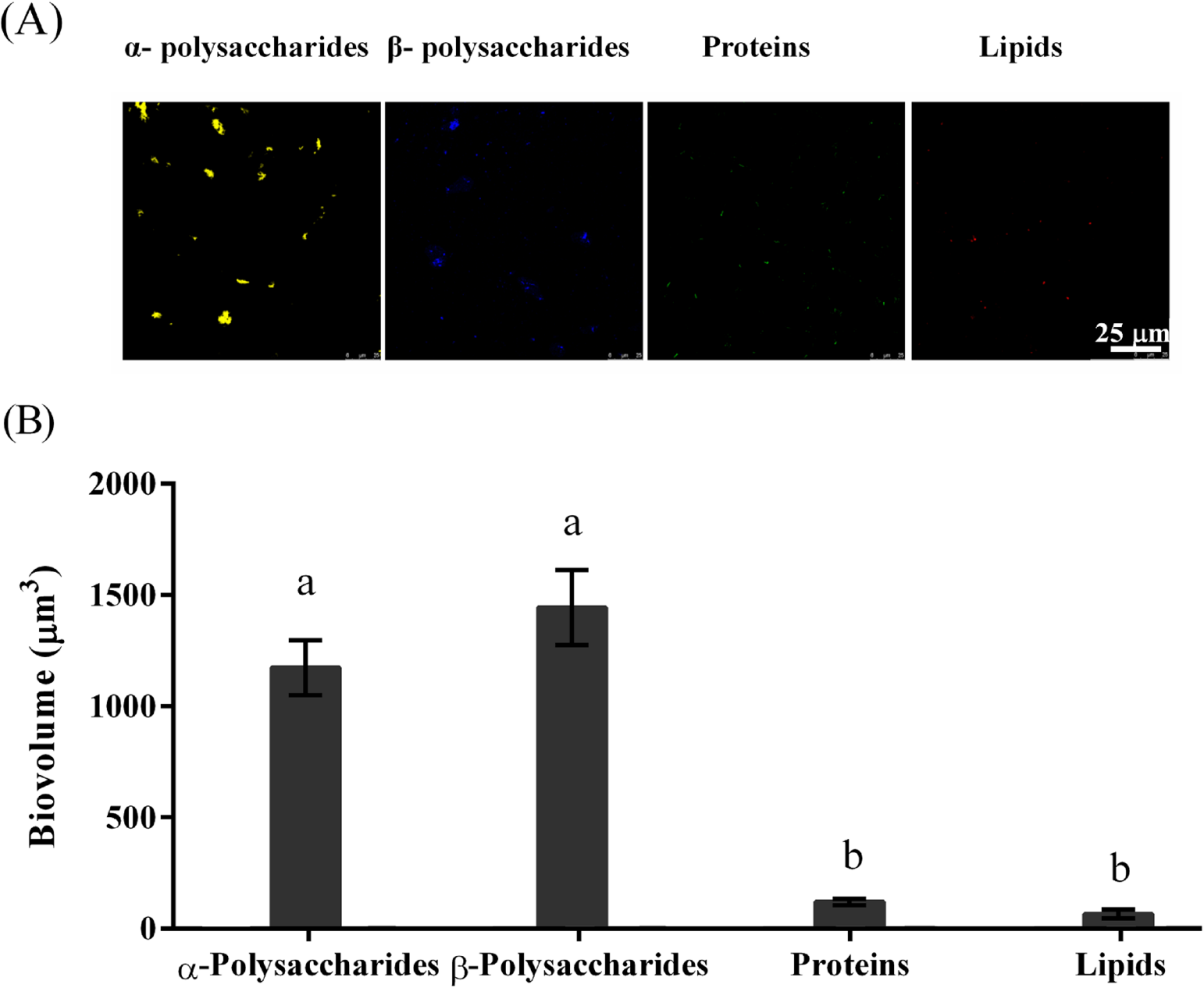
CLSM images (**A**) and biovolumes (**B**) of biofilm extracellular polymeric substances of *Virgibacillus* sp. 1 BFs. Alpha-polysaccharides were combined with Concanavalin A, tetramethylrhodamine conjugate; β-polysaccharides were combined with calcofluor white M2R; proteins were combined with fluorescein isothiocyanate isomer I; lipids were combined with DiIC18(5) oil, 1,1′-dioctadecyl-3,3,3′,3′-tetramethylindodicarbocyanine perchlorate.

The distribution of deep-sea bacterial BFʼs EPS was shown in Fig. 7A, and extracellular polysaccharides were more distributed than proteins and lipids. In Fig. 7B, The *Virgibacillus* sp. 1 showed high biovolumes (*p* < 0.05) in α-polysaccharides and β-polysaccharides in comparison to proteins and lipids. The α-polysaccharide biovolume of *Virgibacillus* sp. 1 BFs was 1174.5 ± 123.6 μm^3^, and the β-polysaccharide biovolume of *Virgibacillus* sp. 1 BFs was 1444.2 ± 167.7 μm^3^. The results suggested that extracellular polysaccharides were main components of extracellular products of *Virgibacillus* sp. 1 BF.

## Discussion

The interaction between offshore bacteria and invertebrateʼ recruitment has been widely acknowledged for many years^17–19^. However, little progress has been made in the relationship between deep-sea bacteria and larval settlement. The present study firstly demonstrated that different deep-sea bacteria could successfully trigger *M. coruscus* larvae to finish planktonic-sessile transition and no mortality was observed.

Some studies have found that the settlement and metamorphosis of marine benthic animals including *Mytilus gallorpvincialis*^8^, *Hydroides elegans*^20^ and *Pocillopora damicornis*^21^ were influenced positively by cell density of offshore bacteria. Other studies also showed that cell density of offshore bacteria was negatively correlated with larval settlement of *Bugula neritina*^22^ and *Balanus amphitrite*^23^. The present study demonstrated that deep-sea bacterial strains exhibited different relationship between cell density and inductive efficiency.

The inducing active strains from the deep sea existed in different genera in this study. The deep-sea bacteria *Virgibacillus* sp. 1 and *Bacillus* sp. 13 belonged to the same genus, but the inductive effect was significantly different; *Pseudoalteromonas* sp. 31 and *Pseudomonas* sp. 1 had no significant difference in post-larvae rate, but they were not the same genus. The species of the deep-sea bacteria may not be related to the ability to induce mussel settlement. This is the same as the previous results for *P. damicornis* larvae^21^, and the specific reasons need further study.

Formalin can kill the bacteria of the BF without damaging the chemical substances on the surface of BFs, suggesting that the surface chemicals of the BF had an effect on larvae^24,25^. Treatments were conducted to determine whether bacterial inductive cues were susceptive to solvents and heat treatment^8,11^. In present study, the inductive efficiency and cell survival rate of treated deep-sea *Virgibacillus* sp. 1 BF significantly reduced. It was consistent with previous studies on the offshore bacteria^11,26^. Treated BFs (100H, 10A and FF) without alive bacteria showed low inducing activity, indicating that the living deep-sea bacteria is necessary to produce chemical signals to promote the settlement process in this species. Previous studies have also demonstrated that the settlement process of *M. coruscus* ^10–13^ larvae also required BFs of living offshore bacteria, as well as *M. galloprovincialis*^8^ and *H. elegans*^20,24^ larvae.

Hadfield^27^ reviewed that *H. elegans* larvae crawled before settlement in the present of offshore bacteria, while larvae in natural seawater swam in a straight line before crawling phase. Bao et al.^25^ found that CW of offshore bacteria could affect *M. galloprovincialis* larval crawling behavior, but it could not induce metamorphosis. Yang et al.^11^ also found that CW of the offshore bacteria could not induce *M. coruscus* larval metamorphosis, but triggered larval settlement behavior. The CW collected from deep-sea *Virgibacillus* sp. 1 BFs triggered *M. coruscus* settlement and metamorphosis, which indicated that release soluble metabolites act on metamorphosis transition.

In present study, the formalin-treated BFs of the deep-sea bacterium *Virgibacillus* sp. 1 had inducing activity, and the synergy between CW and FF can significantly induce larvae to metamorphose into post-larvae, indicating that both factors may be indispensable in metamorphosis. It was consistent with previous research results on the offshore bacterial (*Alteromonas* sp. 1) induction in *M. galloprovincialis*^25^. With the increase of CW concentration, the inducing activity of formalin fixed deep-sea *Virgibacillus* sp. 1 BFs added with CW first increased and then decreased, indicating that there may be an optimal concentration of CW and inhibition of high concentration. A similar result was found in the offshore *Shewanella* sp. 1 BFs induced *M. coruscus* metamorphosis through the synergy between CW and FF^11^.

The BF was composed of bacteria and their metabolites^19,28–31^, and EPS were a key factor for mature BFs formation^30–33^. The BFʼs EPS are mainly composed of three major categories of proteins, polysaccharides and lipids^27,28,30,32,33^. In the present study, the types and contents of extracellular polysaccharides, lipids and proteins produced by the deep-sea bacterial strains *Virgibacillus* sp. 1 were different. The EPS analysis showed that the extracellular polysaccharides (α-polysaccharides and β-polysaccharides) were higher distributed in these the deep-sea bacterium *Virgibacillus* sp. 1 than proteins and lipids. In contrast, the contents of β-polysaccharides and lipids in the BFs formed by offshore bacterial strain *Pseudoalteromonas marina* were lower than α-polysaccharides and proteins. This indicates that EPS matrix may be dependent on the bacterial species^13^.

In conclusion, all tested deep-sea bacteria exhibited inductive activity on the larvae of *M. coruscus*. The living deep-sea bacteria is necessary to produce chemical signals to promote the settlement process in this species. The extracellular polysaccharide may be the one of important signals for planktonic-sessile transition of *M. coruscus*. Two chemical signals that are involved in planktonic-sessile transition, might be a common mechanism in marine mussel *Mytilus* irrespective of the deep-sea or offshore bacteria. Thus, this study of deep-sea bacteria has potential for the application in the mussel seed production and provides novel insight to clarify the bacteria-mussel interaction.

## Materials and methods

### Isolation of bacteria

The deep-sea bacteria were obtained from seawater and sediments following the modified methods^3,11,34^. Seawater and sediments were collected from deep sea (water depth ranging from 1000 to 8900 m) during the period between March 2013 and January 2016. Briefly, one mililiter of bacterial suspension was coated on Zobell 2216E plates with 37 °C incubation in dark for 48 h. Single bacterial colony was selected, purified by repeated streaking on a plate to separate and obtained a pure strain. Pure strains were mixed 1:1 with 0.9% NaCl containing 30% glycerol and stored at − 80 °C.

### Identification by 16S rDNA gene sequences

The preserved bacteria were coated on the Zobell 2216E plate, and the lines were drawn to obtain the single colony. Single colonies were selected and inoculated into Zobell 2216E liquid for extended culture. One milliliter of bacterial solution was used for DNA extraction according to the kit (Shanghai Biocolor Bioscience and Technology Company). PCR primers for the 16S rDNA amplification were 27F and 1492R^35^. The amplification products were sequenced by Shanghai Sangon Biotech Co. Ltd tested products, and the sequenced were blasted at NCBI to determine the bacterial genus.

### Sequence alignment and phylogenetic analyses

The phylogenetic tree construction was performed by reference to the method of Yang et al.^11^. The sequences obtained by the company were analyzed with the sequence of their related in the MEGA software (version 5.05) ClustalW program. The phylogenetic relationship between bacteria was analyzed using a neighbor joining of MEGA software. *Escherichia coli* EC096/10 (Accession No. AONF01000005.1) was obtained from the NCBI GenBank database as an outgroup sequence.

### Spawning and larval culture

Wild adults of *M. coruscus* (2-years-old) were obtained from Gouqi island (122° 44′ E; 30° 73′ N), Zhejiang, China. Fertilization methods refer to Yang et al.^11,36,37^. Spawning mussels were placed in a beaker containing filtered seawater (FSW, 1.2-μm pore size). Fertilized eggs were obtained by mixing sperms and eggs at 18 °C for 20 min and washed with FSW through a nylon plankton net (20 μm) to discard surplus sperms. The swimming D-larvae were obtained after 2 days. Larvae were cultured at the density of 5 larvae ml^−1^, the seawater was changed every 2 days. Larvae developed to pediveliger stage were used to the settlement bioassay (Fig. S2).

### Formation bacterial BFs

The BF formation was performed as a published method^11^. A single colony was picked into 80 ml Zobell 2216E broth and cultured in dark. Bacterial cells were washed and collected through centrifugation three times (3500 rpm, 15 min). These cells were re-suspended to 50 ml autoclaved filtered seawater (AFSW) and made into a suspension. The bacterial suspension was diluted to four initial densities (1.0 × 10^8^, 3.0 × 10^8^, 5.0 × 10^8^, 1.0 × 10^9^ cells ml^−1^) with AFSW. BFs formed in the Petri dish with 20 ml bacterial suspension and one glass slide. Twelve replicates were set for each density. All Petri dishes were placed at 18° C for 48 h in dark to form the BF.

### Preparation of treated BFs of the deep-sea bacteria *Virgibacillus* sp. 1

BFs of *Virgibacillus* sp. 1 showing the highest inducing activity were treated as a modified method of Yang et al.^11^. Formalin solution (5%) was used to immerse BFs for 24 h (FF); 1 h of UV (5 W m^−2^) irradiation was used as UV-treatment; 10% (10E) and 100% (100E) of ethanol solution was used to immerse BFs for 30 min; BFs were heated at 40 °C (40H) and 100 °C (100H) for 30 min^11,13,25,26^. Antibiotic-treatment (0.1A) indicates immersing BFs in the solutions of streptomycin sulfate (1 mg l^−1^) and penicillin G potassium (1 mg l^−1^) for 2 h^38^. Antibiotic solutions of treatments of 1A and 10A were 10 and 100 times that of 0.1A, respectively.

### Larval settlement bioassays

Larval bioassays were conducted as the previously published method^11^. Twenty pediveliger larvae, a glass slide with BF or treated BF and 20 ml AFSW were placed in a petri dish. The percentages of post-larvae were recorded until 96 h. Of these, the percentages of post-larvae at 48 h were used to assess the inducing activity of bacterial BFs. Post-larvae were observed using an Olympus (BX51) stereoscopic microscope. A clean sterilized slide was blank groups. Nine replicates were set for each group. All experiments were completed at 18 °C.

### The inducing activity of conditioned water (CW) of *Virgibacillus* sp. 1 BFs

CW of *Virgibacillus* sp. 1 BFs was collected according to Yang et al.^11^. Five milliliters of cell suspension, 15 ml AFSW and a sterilized slide were added to sterile Petri dish and cultured for 48 h under dark conditions. The obtained solution filtered by 0.22 μm was served as 100% CW. Similarly, 5 ml bacterial suspension was added into Perti dish containing 35, 5, and 1.7 ml of AFSW to prepare 50% CW, 200% CW and 300% CW, respectively. To investigate the synergy between CW and FF, different concentrations of CW and FF were put together in a sterile Petri dish. Twenty milliliters of CW (50%, 100%, 200% and 300%), and 20 larvae and FF were added into Petri dish to conduct settlement and metamorphosis bioassay. Nine replicates were set for CW at different concentrations.

### Cell survival of deep-sea bacteria

Survival of deep-sea bacterial BFs was performed according to Bao et al.^39^. The *Virgibacillus* sp. 1 BFs were stained with 4 mM 5-cyano-2,3-ditolyl tetrazolium chloride solution in dark, washed after 4 h, and stained through 1 μg l^−1^ of 4′,6-diamidino-2-phenylindole at 25 °C for 5 min. Ten fields were randomly selected for counting using an Olympus microscope (magnification: 1000 ×). Three replicates were set.

### Cell density of deep-sea bacteria

Cell density was counted following the methods^11,40^. The *Virgibacillus* sp. 1 BFs fixed in 5% formalin solution (FF) were washed and stained using 0.1% acridine orange for 5 min. Samples were observed under epifluorescence microscope (Olympus BX51) at 1000 × magnification, and 10 random fields were selected for counting. Three replicates were set for each bacterial density to determine the density of the BF formed at different initial concentrations.

### Confocal laser scanning microscopy (CLSM) and image analysis

Extracellular products were stained according to the method of Liang et al.^12^. *Virgibacillus* sp. 1 and *Pseudoalteromonas* sp. 33 BFs were washed three times with 0.9% saline and stained with corresponding dye for 20 min no light. Slides were washed three times again and observed in CLSM (Leica TCS SP8, Germany). Three parallels were set for each bacterial. Three fields were randomly selected for each BF, and nine different fields of view were used for imaging and analysis. α-polysaccharides were combined with Concanavalin A, tetramethylrhodamine conjugate (conA-TMR); β-polysaccharides were combined with calcofluor white M2R (CFW); proteins were combined with fluorescein isothiocyanate isomer I (FITC); lipids were combinedwithDiIC18(5) oil, 1,1′-dioctadecyl-3,3,3′,3′-tetramethylindodicarbocyanine perchlorate (DiD'oil). Each CLSM image was acquired by the LAS X Version (pixels: 1024 × 1024, z-step: 0.20 μm). The quantification of each component was conducted by Image J software. The threshold value of CLSM image was used to calculate the biovolume (μm^3^) of each component.

### Data analysis

Data expressed in percentages were arcsine transformed. Experimental data were analyzed by JMP software (ver. 10.0.0). Correlation analysis was performed via Spearman's rank correlation test. Kruskal–Wallis followed by the Steel–Dwass All Pairs test was conducted to determine significant variance.

## Supplementary Information

Supplementary Information.
