# Supplementary material for: Deep-sea bacteria trigger settlement and metamorphosis of the mussel Mytilus coruscus larvae
Source: Sci Rep. 2021 Jan 13;11:919. doi: 10.1038/s41598-020-79832-8 (PMC7806842; doi:10.1038/s41598-020-79832-8)
Supplement: Supplementary file 1 — Supplementary Information. [file 41598_2020_79832_MOESM1_ESM.docx]

**Deep-Sea Bacteria Trigger Settlement and Metamorphosis of the Mussel *Mytilus coruscus* Larvae**

Rui-Heng Chang^1, 2*^, Li-Ting Yang^1, 2*^, Ming Luo^3*^, Yihan Fang^1, 2^, Li-Hua Peng ^1, 2^, Yu-Li Wei ^1, 4^, Jia-Song Fang ^1, 4^, Jin-Long Yang^1, 2†^, Xiao Liang^1, 2†^

*^1^ International Research Center for Marine Biosciences, Ministry of Science and Technology, Shanghai Ocean University, Shanghai, China*

*^2^ Southern Marine Science and Engineering Guangdong Laboratory, Guangzhou, China*

*^5^ Hainan Academy of Ocean and Fisheries Sciences, Haikou, China*

*^4^ Hadal Science and Technology Research Center, Shanghai Ocean University, Shanghai 201306, China*

Running head: Deep-sea bacteria promote mussel settlement

^†^ Corresponding author. E-mail: jlyang@shou.edu.cn, x-liang@shou.edu.cn

Tel: + 86-21-61900403; Fax: + 86-21-61900405

^*^ These authors contributed equally.

**Figure S1.** CLSM images of biofilm extracellular polymeric substances (polysaccharides, proteins and lipids) of two deep-sea bacterial species. (A) and (B) represent two replicates.

**Figure S2.** The mussel pediveligers (A) and post-larvae (B) compare photos.
